# Effects of Zinc Oxide Nanoparticles on Physiological and Anatomical Indices in Spring Barley Tissues

**DOI:** 10.3390/nano11071722

**Published:** 2021-06-30

**Authors:** Vishnu D. Rajput, Tatiana Minkina, Aleksei Fedorenko, Natalia Chernikova, Tara Hassan, Saglara Mandzhieva, Svetlana Sushkova, Vladimir Lysenko, Mikhail A. Soldatov, Marina Burachevskaya

**Affiliations:** 1 Academy of Biology and Biotechnology, Southern Federal University, 344090 Rostov-on-Don, Russia; tminkina@mail.ru (T.M.); afedorenko@mail.ru (A.F.); nat.tchernikova2013@yandex.ru (N.C.); tara.hassan@su.edu.krd (T.H.); msaglara@mail.ru (S.M.); terra_rossa@mail.ru (S.S.); vs958@yandex.ru (V.L.); marina.0911@mail.ru (M.B.); 2 The Smart Materials Research Institute, Southern Federal University, 344090 Rostov-on-Don, Russia; mikhail.soldatov@gmail.com

**Keywords:** cellular uptake, cytotoxicity, stomata, trichomes, chlorophyll fluorescence, elemental analysis

## Abstract

The aim of the present work was to investigate the toxic effects of zinc oxide nanoparticles (ZnO NPs, particle size < 50 nm) on the physiological and anatomical indices of spring barley (*Hordeum sativum* L.). The results show that ZnO NPs inhibited *H. sativum* growth by affecting the chlorophyll fluorescence emissions and causing deformations of the stomatal and trichome morphology, alterations to the cellular organizations, including irregularities of the chloroplasts, and disruptions to the grana and thylakoid organizations. There was a lower number of chloroplasts per cell observed in the *H. sativum* leaf cells treated with ZnO NPs as compared to the non-treated plants. Cytomorphometric quantification revealed that ZnO NPs decreased the size of the chloroplast by 1.5 and 4 times in 300 and 2000 mg/L ZnO NP-treated plants, respectively. The elemental analysis showed higher Zn accumulation in the treated leaf tissues (3.8 and 10.18-fold with 300 and 2000 mg/L ZnO NPs, respectively) than the untreated. High contents of Zn were observed in several spots in ZnO NP-treated leaf tissues using X-ray fluorescence. Deviations in the anatomical indices were significantly correlated with physiological observations. The accumulation of Zn content in plant tissues that originated from ZnO NPs was shown to cause damage to the structural organization of the photosynthetic apparatus and reduced the photosynthetic activities.

## 1. Introduction

The application of nanoparticles (NPs) in agriculture was accepted at the beginning of the 21st century [1], and more than 231 products are available for various agricultural applications (https://product.statnano.com/ (accessed on 15 March 2021)). There has been rising interest in the application of NPs to reduce the dependence on chemical fertilizers for sustainable crop production and food security to meet the needs of nutritional requirements of the rapidly growing global population [2,3]. Zinc-based NPs are among the most widely used NPs in the nanoindustry [4], and are produced (550–5550 t a^−1^) 10 to 100 times more than the other NPs [5,6]. They are extensively utilized in the cosmetics industry [7], medicines [8], food, and solar cells [9], which inevitably leads to their transfer to the environment [10]. The increased application and continuous release undoubtedly lead to ZnO NPs accumulating in the ecosystem. Due to their ability to absorb and accumulate pollutants, plants play a crucial role in this transfer, especially in the food chain [11].

The application of ZnO NPs in agriculture has indicated both positive and negative impacts on plant growth [12,13,14,15]. The foliar application of 10 mg/L ZnO NPs expressed positive impacts on chlorophyll, phosphorus, and protein contents in *Cyamopsis tetragonoloba* [16]; 160 mg/kg promoted the seed yield of *Glycine max* [17], 8 mg/L improved the enzymatic activities of *Lycopersicon esculentum* [12,18] and boosted the plant defense system [19]. The treatment of *Triticum aestivum* with ZnO NPs (250–2000 mg/L) increased the chlorophyll and protein contents [20]. However, seed treatment with ZnO NPs at a concentration of 300 mg/L reduced growth by 80% and the chlorophyll content by more than 50% in *Arabidopsis thaliana* [21]. The reduced root and shoot length of *Raphanus sativus* and *Spinacia oleracea* treated with ZnO NPs was also noted [22,23]. The toxic effect on the growth and development of the *A. thaliana* plants was also revealed when exposed to lower concentrations of ZnO NPs [24,25]. In another study, both positive and negative effects were shown in *Stevia rebaudiana*, depending on the concentration of applied ZnO NPs, [14]. Based on these studies, the response of plants to Zn-based NPs is thought to depend on the plant species, type and size of the NPs, and the applied concentration.

Once NPs enter the plant cells, such as the vascular bundle (xylem) and stele, they reach the aerial parts [26] through cellular pores [2,27] via apoplast or symplast pathways [4] and interact with cellular and sub-cellular organelles. During this penetration and translocation process, it could damage the sub-cellular organization [28] and may affect photosynthesis [29,30,31]. It is evident from recent studies that Zn may be accumulated in plants in their tissues and cellular and sub-cellular organelles, such as chloroplasts, cell membranes, vacuoles, nuclei [32,33], and modulate cellular organizations [28,34]. The high accumulation of Zn has also been reported in plant tissues by various recent findings performed with Zn-based NPs in pots and hydroponic conditions [13,23,35,36,37,38].

The studies dealing with the toxic effects of ZnO NPs on the organelles of plant cells, especially chloroplasts, and their correlation with the function of photosynthesis are scarce. Their effect on photosynthetic machinery is also poorly known. Therefore, to fill this gap, the present study was designed to explore the effects of ZnO NPs on the aerial parts of spring barley (*Hordeum sativum* L.) under hydroponic conditions. We hypothesized that the exposure to ZnO NPs would not only negatively affect the chloroplast organization but also affect the chlorophyll fluorescence and elemental distribution in the leaf cells. The present investigation could be helpful in minimizing the hazardous impact, if any, associated with field applications of metal-based NPs, especially ZnO.

## 2. Materials and Methods

### 2.1. Experimental Set-Up

#### 2.1.1. Nanoparticle Preparation and Characterization

Commercial grade zinc oxide (ZnO) NP powder (particle size < 50 nm) was purchased from Sigma-Aldrich, St. Louis, MO, USA. The purchased NPs were used as received, without further changes. The NPs were poured into double distilled water to prepare the desired concentration. To achieve a well-mixed dispersion and to minimize aggregation, the NP solution was shaken and ultra-sonicated (stabilization step) before application in the batch experiments. The properties of the ZnO NPs were characterized by transmission electron microscopy (TEM; Tecnai G12, FEI Company, Czech Republic), powder X-ray diffraction was used to estimate the crystalline sizes, and their stability was analyzed using dynamic light scattering (DLS) and ζ-potential measurements.

##### Characterization of ZnO Nanoparticles by Transmission Electron Microscopy (TEM)

Examination of the ZnO NP samples by TEM was carried out for two different samples. (1). The nanoparticles were applied to a grid substrate in a dry powdered form. (2). The nanoparticles were diluted in water in the form of suspension with a concentration of 300 mg/L, and then a drop of the suspension was placed on a support film and dried at room temperature.

##### Powder X-ray Diffraction of ZnO Nanoparticles

The powder X-ray diffraction data were collected on a D2 Phaser (Bruker Corp., Billerica, MA, USA) using Cu Kα radiation (*λ* = 0.1541 nm). The Scherrer formula was used for the determination of the mean crystalline size, as follows:

(1) $$d=\frac{K\times \lambda }{cos\theta \times b}$$

where *d* is the mean crystalline size, *K* is the Scherrer constant for spherical particles, *λ* is the wavelength of the radiation, *cosθ* is the Bragg angle, and *b* is the additional contribution to the integral broadening in radians.

##### Hydrodynamic Dynamic Light Scattering (DLS) and ζ-Potential Analysis of ZnO Nanoparticles

The hydrodynamic sizes of the particles in the colloidal samples and their stability were analyzed using dynamic light scattering (DLS) and ζ-potential measurements. The DLS measurements were performed using backscatter geometry with a 780 nm laser on a Nano-flex (Microtrac, Montgomeryville and York, PA, USA). For each sample, a set of five 2 min scans was collected. The ζ-potential data was collected using a Stabino (Particle Metrix GmbH, Ammersee, Germany).

#### 2.1.2. Plant Growth and Performance

The seeds of the widely cultivated *H. sativum* distichum cv. Travnik were visually checked for any morphological damage, and 25 healthy seeds were selected, sterilized, and placed at an equal distance over a round piece of filter paper in clean Petri dishes with a diameter of 95 mm. Then, 5 mL of distilled water with (300 and 2000 mg/L ZnO NPs) or without ZnO NPs (0 mg/L) was applied to the Petri dish containing no additional nutrients and maintained in a growth chamber at 28 °C. All treatments were performed in triplicate. In each vessel, 10 germinated seeds were transferred to plastic vessels (L × W × H: 100 × 60 × 50 mm) (in triplicate) in 50 mL of modified half-strength of Hoagland’s solution (0.2% KNO_3_) with (300, 2000 mg/L ZnO NPs—treatments) or without NPs (0 mg/L—control) and maintained in the growth chamber at 25 ± 2 °C with a 16 h light (with a 36 W/635 fluorescent lamp, illumination of 4–5 kL) and 8 h dark cycle for 2 weeks. The pH of the aqueous solution was adjusted to 6.0 ± 0.02.

### 2.2. Elemental Analysis in Plant Tissues

#### 2.2.1. Non-Destructive Method

Elemental analysis was performed using non-destructive X-ray fluorescence spectroscopy (XRF). The middle leaves were collected from 2-week-old *H. sativum* seedlings and processed (leaves were dried at room temperature and the surface was flattened using coverslips) for XRF. Images of the areas for XRF analysis were collected using an optical microscope (Mikmed-6, JSC “Lomo”, St. Petersburg, Russia). The XRF spectra were measured using a microfocus spectrometer M4 Tornado (Bruker Corp., Billerica, MA, USA). The X-ray tube was operated at 50 kV and 600 µA, and the sample analyses were done in a vacuum in order to have better statistics for light elements (Si, P, S, Cl). The data were collected from two different areas for each sample (for the control sample, the XRF spectra were collected from three areas) and averaged for 200 points in each area.

#### 2.2.2. Destructive Method

The determination of Zn uptake and accumulation in 2-week-old aerial plant tissues was performed by the combustion method at 450 °C. An air-dried 1 g sample was used, and the ash was dissolved in 5 mL of 20% HCl and filtered through 0.45 µm Whatman filter paper. The nanoparticle concentration was determined using an atomic absorption spectrophotometer (AAS) (KVANT 2-AT, Kortec Ltd., Moscow, Russia) with a wavelength diapason of 190–900 nm at room temperature.

### 2.3. Physiological Indices

#### 2.3.1. Photosynthetic Effectiveness Evaluating by Fluorescence Kinetic Parameters

The potential ability of ZnO NPs to influence the photosynthetic processes was evaluated by the photoinduction curves using a previously described method of chlorophyll fluorescence video registration [39]. Imaging of the chlorophyll fluorescence was performed using an ultrahigh sensitive video camera (VNC-748-H3, EVS, Moscow, Russia) equipped with an interference filter operating at 690 nm with a half-band width of 5 nm (Esco Products, Oak Ridge, NJ, USA). After 20 min of dark adaptation, chlorophyll fluorescence was excited by two 5 W blue (λ_max_ = 470 nm) photodiodes applied with an incident actinic light of photosynthetic photon flux density (PPFD) of 120 µmol m^−2^ s^−1^ at the adaxial leaf surface.

A sequence of images was retrieved from the video file using the ImageJ program. Photoinduction curves were obtained for the regions of interest (ROI) selected at the leaf surface using the same program. Each photoinduction curve was used for the calculation of the fluorescence decrease ratio.

Rfd = (Fp − Fs)/Fs(2)

where Fp is the peak (maximal) fluorescence detected within the photoinduction curve, and Fs is the steady-state fluorescence detected at the end of actinic light illumination.

#### 2.3.2. Measurement of Stomatal Aperture and Trichome Morphology

Fresh leaf samples were obtained and prepared for scanning electron microscopy (SEM) to observe the stomatal aperture and trichome morphology. The leaf samples were exposed to sunlight for 30 min and then frozen at −45 °C and lyophilized by a Freeze Dryer LZ-9 “Frigera Ltd.,” Brno–Chernivice, (Czech Republic). The adaxial surface was sprayed with a gold/palladium target using a Mini Sputter Coater SC7620 vacuum deposition unit (Quorum Technologies, Laughton, East Sussex, UK) with a 30 s spraying mode at 18 mA. The samples were analyzed using an EVO-40 XVP SEM, Carl Zeiss, Oberkochen, Germany at an accelerating voltage of 10–15 kV. The stomatal density was counted by encircling the similar area of all the samples and presented as average. The stomatal openings and trichome areas were measured by ImageJ software.

### 2.4. Anatomical Indices

#### 2.4.1. Cellular and Sub-Cellular Structural and Ultrastructural Observations

The preparation of plant tissue samples for cellular structural and ultrastructural observation was performed according to the method developed by Fedorenko et al. [40] for light and TEM observations. A 1 mm leaf sample was obtained from the middle of a fresh leaf directly taken from the experimental plants. The collected samples were fixed using 2.5% glutaraldehyde/0.1 M phosphate buffer solution (PBS) at room temperature for 2 h. The fixed samples were washed with PBS. After washing, the samples were incubated for 1 h in 1% OsO_4_/0.2 M PBS solution. The increasing concentration of ethanol and acetone (separately 50%, 70%, 96%, and 100%) were used for dehydration. The dehydrated samples were embedded in Epon-812 embedding medium. Semi-thin (about 1 μm thick) and ultrathin (about 100 nm thick) sections were prepared by a microtome (Leica EM UC6, Leica, Wetzlar, Germany). The semi-thin sections were stained with 1% toluidine blue and examined under the light optical microscope (Mikmed-6 St. Petersburg, Russia). The ultrathin sections were stained with a lead citrate contrast agent and examined by TEM (Tecnai G12, Tecnai G12 spirit biotwin, FEI Company, Czech Republic).

#### 2.4.2. Cytomorphometric Quantification

The cytomorphometric quantification of the sub-cellular regions (chlorenchyma cell area, ratio of chlorenchyma cell of total leaf cut area, stomata, and trichome density) was measured by ImageJ software considering 10 images obtained from different sites of each sample. The results were calculated and presented as average values with standard error (S.E.) using one-way ANOVA.

### 2.5. Statistical Analysis

Statistical analysis was performed using Microsoft Excel 2016 and SPSS- Statistics 19.0 Pack-1 software (Chicago, IL, USA). All the data presented is an average with standard error (S.E.) using one-way ANOVA. Statistical significance was determined using Fisher’s least significant difference (LSD) test. Differences were considered significant at *p* ≤ 0.05. For the data obtained, the levels of difference were statistically significant. For elemental analysis (in triplicate), the induction curve (the average of 8 replicates), and cytomorphometric estimation, 10 images from different locations of the same samples in triplicate were considered.

## 3. Results

### 3.1. Characterization of ZnO Nanoparticles, Crystalline Sizes, Hydrodynamic Sizes of the Particles and Their Stability in Colloidal Solutation

**Figure 1:**
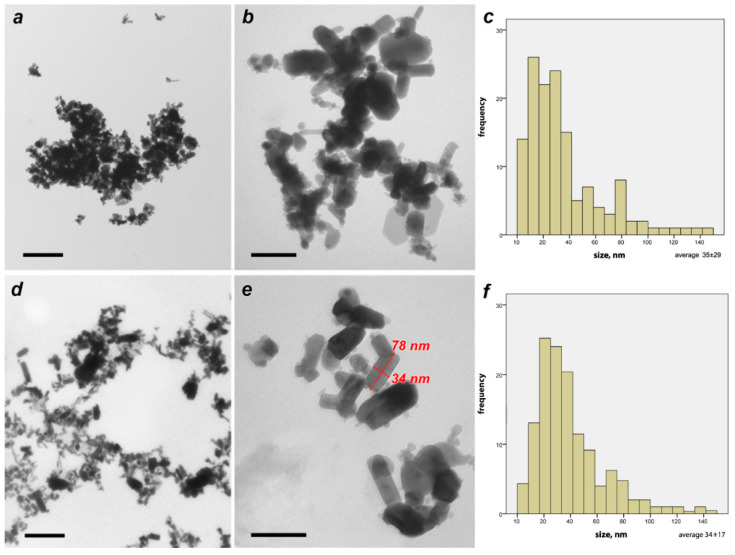
Zinc oxide nanoparticles: TEM images indicate particle size (**a**,**b**) powder form, (**d**,**e**) 300 mg/L, and (**c**,**f**) show particle size distribution and size distribution frequency of powder and 300 mg/L ZnO NP, respectively. Scale bar: (**a**,**d**)—400 nm, (**b**,**e**)—100 nm.

The TEM observations showed that the NPs consisted of hexagonal lamellar particles with a major axis of 50–150 nm and a minor axis of 20–70 nm (Figure 1a,b). In the first variant, a large fraction of the particles stuck together into loose conglomerates 1000–2000 nm in size (Figure 1b). In the 300 mg/L ZnO NP colloidal solution, the particles were more scattered. The range of particle size was from 10 to 150 nm. The highest frequency of the investigated particle sizes ranged from 15 to 40 nm (Figure 1c,f). The measurements were carried out by measuring the smallest axes of all particles that fell into a random field of view of the microscope in 10 replicates.

**Figure 2:**
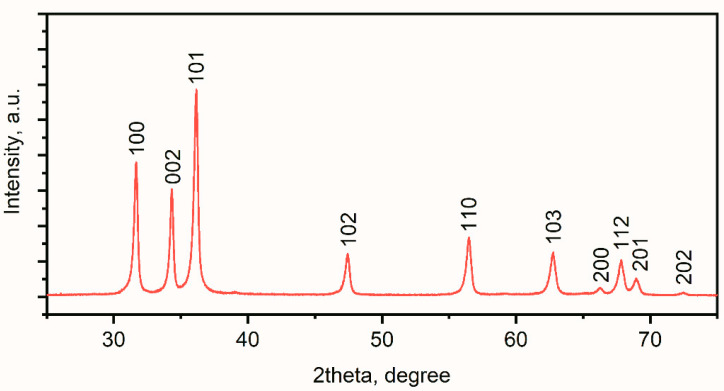
X-ray diffraction pattern recorded for ZnO nanoparticles.

The 2θ values corresponding to 100, 002, 101, 102, 110, 103, 112 and 201 reflections were used for ZnO XRD patterns to estimate the crystalline sizes (Figure 2). The mean crystalline size for the ZnO NPs was estimated to be 40 nm.

**Figure 3:**
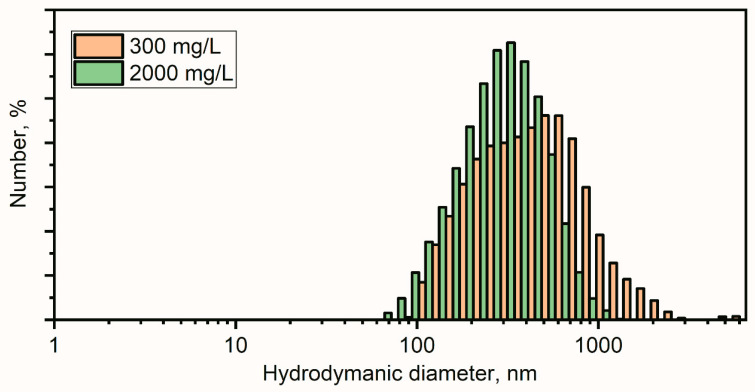
DLS of 300 mg/L and 2000 mg/L colloidal solution of ZnO nanoparticles.

The hydrodynamic particle diameter distribution for aqueous solutions of ZnO NPs showed a stable colloidal solution with means of 360 nm (300 mg/L) and 290 nm (2000 mg/L) (Figure 3). Larger hydrodynamic diameters of the particles in colloidal solution as compared to the crystalline size suggested the aggregate formation of the ZnO NPs. Such aggregates showed moderate stability observed by the ζ-potential measurements of −21 ± 1.3 mV for (for the 300 mg/L concentration) and 6.5 ± 1.2 mV (for the 2000 mg/L concentration).

### 3.2. Zn Accumulation in Above-Ground Tissues of H. sativum

**Figure 4:**
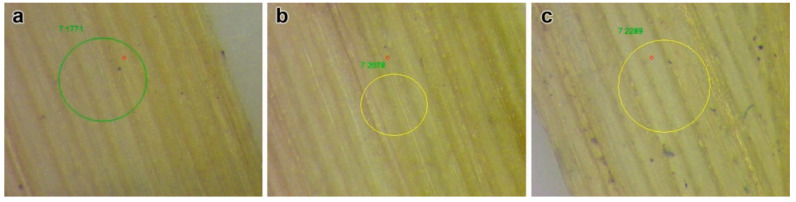
Zinc content of leaves by selecting plots using X-ray fluorescence spectroscopy. Images: (**a**) control, (**b**) 300, and (**c**) 2000 mg/L ZnO NPs.

The destructive methods of Zn analysis showed high accumulations in above-ground tissues. They were 3.8 and 10.3 times higher in 300 and 2000 mg/L ZnO NP-treated plants, respectively, than in the control (Table 1). The XRF analysis for elemental contents showed the presence of Zn in leaf tissues. The high concentrations of Zn in leaf tissues were visible in several spots in ZnO NP-treated leaf samples with respect to the control (Figure 4).

However, for plants treated with 300 mg/L ZnO NPs, the standard deviation was high due to the heterogeneity of the sample. The standard deviation for light elements was also quite high due to the reduced signal from the emission lines of light elements. However, the main interest was the concentration of third transition metals, particularly, Fe, Cu, and Zn. The high concentrations of Cu, Fe, and Zn were visible in several spots in leaf samples treated with 300 mg/L ZnO NPs (Supplementary data: Tables S1 and S2, Figure S2). The leaf samples treated with 2000 mg/L ZnO NPs (Figure S3) had a heightened concentration of Zn, while the Fe and Cu concentrations seemed to be in the order of the control sample (Figure S1).

### 3.3. Effects of ZnO NPs on Physiological Indices

#### 3.3.1. Chlorophyll Fluorescence Kinetics in *H. sativum* Leaves from Plants Treated with Different Concentrations of ZnO NPs

**Figure 5:**
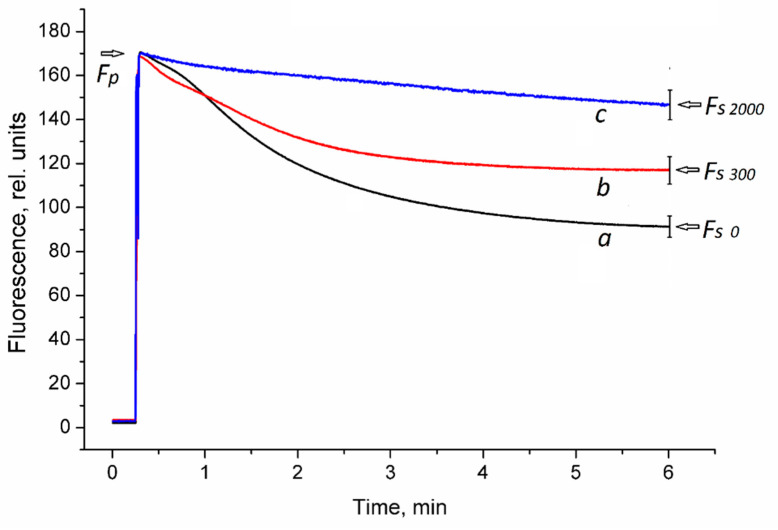
Chlorophyll fluorescence kinetics in *H. sativum* leaves from plants germinated in the presence of different ZnO NP concentrations. Photoinduction curves averaged over 8 repetitions are shown. (**a**) 0 mg/L ZnO NPs (control); (**b**) 300 mg/L ZnO NPs; (**c**) 2000 mg/L ZnO NPs. F_p_—peak (maximal) fluorescence level; F_s_—steady-state fluorescence level. Curves were normalized by F_p_. Fluorescence decrease ratios, R_fd_ = (F_p_ − F_s_)/F_s_, mean ± SD, were R_fd 0_ = 0.87 ± 0.05, R_fd 300_ = 0.45 ± 0.03, and R_fd 2000_ = 0.16 ± 0.02 at *p* = 0.05, *n* = 8.

**Figure 6:**
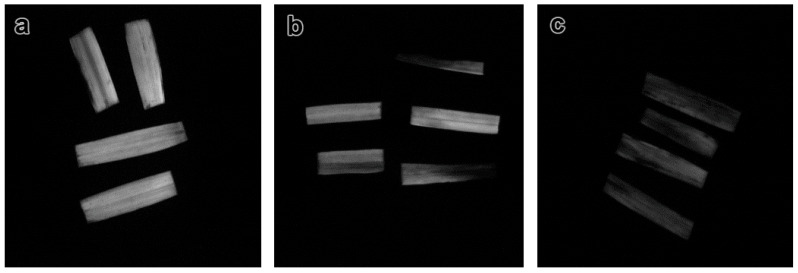
Chlorophyll fluorescence of *H. sativum* leaves by video registration. (**a**) control, (**b**) 300 mg/L ZnO NPs, (**c**) 2000 mg/L ZnO NPs.

The photoinduction curves obtained in the experiments showed that the amplitude of the chlorophyll fluorescence kinetics decreased under the NP treatment (Figure 5). Thus, the Rfd values calculated for the plants treated with 2000 mg/L ZnO NPs were significantly lower than that of plants treated with 300 mg/L ZnO. The Rfd values for the control plants were the lowest. The chlorophyll fluorescence of dark-adapted leaves of *H. sativum* plants was reordered by the video registration method (Figure 6).

#### 3.3.2. Effects of ZnO NPs on Stomata and Trichome Morphology

**Figure 7:**
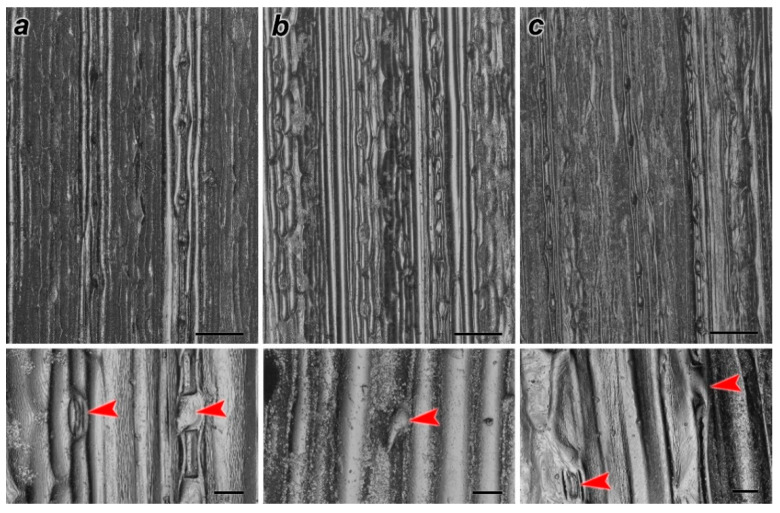
Effects of ZnO NPs of stomata and trichome morphology of *H. sativum*, adaxial surface of leaf showed by sub-figures (**a**) control, (**b**) 300 mg/L ZnO NPs, and (**c**) 2000 mg/L ZnO NPs. Red arrows indicate trichomes and stomata.

The SEM images of the leaf samples showed deformations in the stomata and trichome morphology of treated *H. sativum* leaves (Figure 7). The cytomorphometric quantification results indicated an increase in the area of stomata and trichome in 300 ZnO NP-treated leaves, and it was higher than the control, whereas size reduction was noted for leaves treated at higher concentrations (Table 2).

#### 3.3.3. Effect of ZnO NPs on Leaf Cellular and Sub-Cellular Organelles

**Figure 8:**
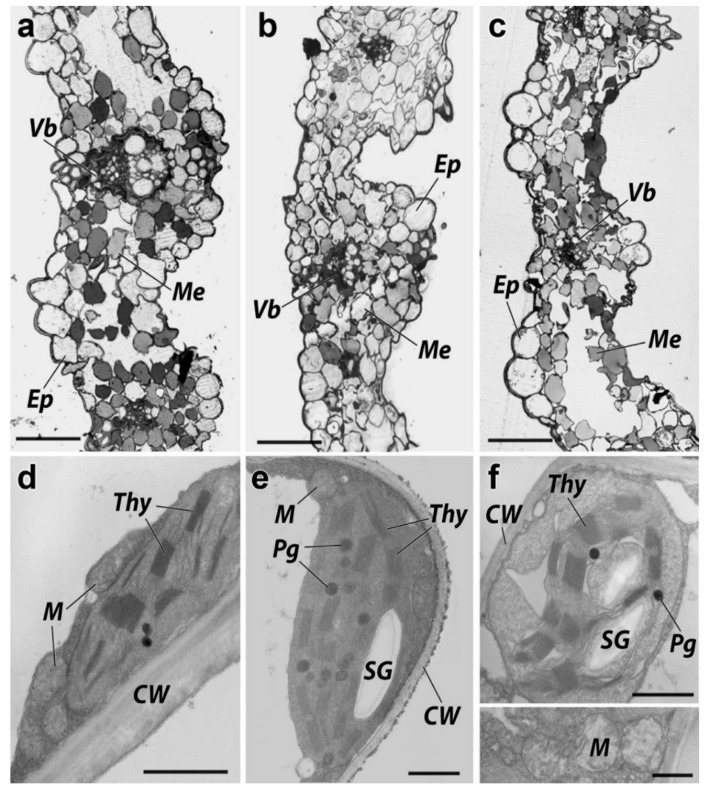
Cross-section of a barley leaf plate: (**a**,**d**) control, (**b**,**e**) hydroponically grown *H. sativum* with 300 mg/L ZnO NPs, (**c**,**d**) hydroponically grown barley with 2000 mg/L ZnO NPs. Ep—epidermis, Me—mesophyll, VB—conductive beam, CW—cell wall, Cl—chloroplast, P—plastoglobule, Thy—thylakoids granules, M—mitochondria, SG—starch grain, V—vacuole. Scale bar: (**a**–**c**) 50 µm, (**d**) 1 µm, (**e**) 0.5 µm, (**f**) 1 µm, (insert) 0.5 µm.

The light optical photograph analysis of the *H. sativum* leaf revealed an anatomical structure typical of monocotyledonous in the control variant (Figure 8a). The structure of the leaf tissue was characterized by an ordered organization and the cell’s uniform localization in the chlorenchyma of the leaf. The division of mesophyll cells into palisade and spongy parenchyma was remotely traced. The cells of the parenchyma are located in several rows between the upper and lower epidermal layers, which were characterized by the round form. The insignificant proportion of tightly contacting cells and the presence of an extensive intercellular space was established for the above-mentioned cells of parenchyma spatial organization.

The light optical photograph analysis of the *H. sativum* leaf tissue grown under ZnO NPs concentrations equivalent to 300 mg/L and 2000 mg/L showed less ordered cell organization in the leaf chlorenchyma. The mesophyll division into columnar and spongy parenchyma, as in the control, was poorly expressed (Figure 2c and Figure 8b). The cells of the parenchyma had a flattened shape along the axis of the leaf plate. The cell spatial organization of the *H. sativum* plants in the variant treated by 300 mg/L ZnO NPs was denser than in the control. The areas of chlorenchyma located between the conducting bundles were strongly flattened. Epidermal cells were smaller and less rounded than in the control samples, on average. The parenchyma was generally denser than in the control, but local large areas of the intercellular space were encountered in the samples of the *H. sativum* grown in 2000 mg/L ZnO NPs.

The main morphometric parameters of *H. sativum* leaf cut cells treated with 300 mg/L and 2000 mg/L ZnO NPs were comparable to the control. The average cell areas of chlorenchyma for variants treated with 300 mg/L and 2000 mg/L ZnO NPs and the control were 131, 146, and 138 μm^2^, respectively. The ratio of chlorenchyma cell area to the intercellular space area were amounted to 0.57, 0.6, and 0.63, and the average numbers of plastids per cell were 6, 5, and 4 in control, and variants treated with 300 mg/L and 2000 mg/L ZnO NPs, respectively (Table 2).

The parenchyma and xylem of the vascular bundles of the leaf had normal ultrastructural characteristics in the control cells of the chlorenchyma (Figure 8a). Chloroplasts were predominantly lenticular in shape, contained numerous granules, and relatively rare plastoglobules were up to 0.1 μm in diameter. Granules, with a number of thylakoids up to 15 units, were evenly distributed over the entire area of the plastid cut. Ellipse-shaped mitochondria contained a dense matrix and teardrop-shaped cristae. Starch grains were absent or very scarce.

Exposure to ZnO NPs caused significant changes in the ultrastructure of *H. sativum* leaf cells. A decrease in electron density and vacuolization of the matrix was noted in the stroma of the chloroplasts (Figure 8e,f). Organelles had a predominantly asymmetric shape, and the contours of the surface membrane in some areas were tortuous. The grains were unevenly distributed over the area of the organelle and were concentrated in the center around the starch grains in the *H. sativum* plants grown under 2000 mg/L ZnO NPs.

The number of thylakoids in the granules reached up to 25–30 units and a single thylakoid of the stroma was enlarged. There was a significant increase in the number of plastoglobules and an increase in their size up to 0.3 μm (Table 3). The mitochondria contained sparse, randomly oriented cristae, a clear matrix, and looked swollen (Figure 8f, insert). The cell walls were thin compared to the control leaf.

## 4. Discussion

Zinc is necessary and the least available micronutrient for plants. The phytotoxic threshold concentration may range between 200–500 mg/kg [41] and vary from species to species within the plant kingdom. In the present study, the hydroponic method was used as it allowed for simplifying the model, where the parameters of plant growth could be easily controlled [42,43]. Concentrations of 300 and 2000 mg/L of ZnO NPs were considered, as concentrations up to 1600 mg/L were identified to stimulate the germination process and the concentration of 3200 mg/L negatively affected the growth of *Allium cepa* [44]. Our results also show a high accumulation of Zn (224.6 ± 17.3 mg/kg in 2000 mg/L-treated above-ground tissues) in the above-ground tissues of *H. sativum*.

A high content of Zn (1200 mg/kg dry weight) was determined in barley leaves grown in pots amended with 30 mmol/kg of Zn in the form of ZnSO_4_, whereas plants treated with ZnO NPs showed higher contents of Zn in the root tissues [45]. During the dissolution of ZnO in the root zone, nanosized particles were reported to be absorbed more readily than larger-sized particles, such as bulk ZnO particles. Near-edge X-ray absorption spectroscopy showed that ZnO NPs of 40 nm were dissolved by roots more easily than their larger-size counterparts [46]. The content of Zn ions determined to be within the tissues of *G. max* treated with 4000 mg/L ZnO NPs was from the NPs, but the X-ray absorption spectroscopy investigation failed to figure out the exact source of these Zn^2+^ [47]. Thus, the comparative analysis showed that the amount of Zn accumulated in the roots versus what actually translocated into the above-ground tissues of plants could be an extended depiction of Zn that is adsorbed rather than absorbed by root tissues [45]. The mechanism by which Zn ions are liberated from NPs has yet to be fully elucidated. Due to their small size, NPs can penetrate plant cell walls, enter the apoplast, and travel to the aerial part. The processes of Zn uptake and partitioning in plant tissues are highly controlled [15,48].

The toxicity of the dissolved Zn may be mainly related to the greater availability of this zinc form for plant roots. It was reported that the toxic effects of ZnO NPs on *Brassica napus* were due to the dissolution of Zn^2+^ ions [49], although the high concentrations of Zn in plant tissues could decrease the chlorophyll content, disturb chloroplast organization, and reduce the number of thylakoid and grana [50]. It was reported that NPs could interfere with water and nutrient transport in the above-ground tissues of plants [51].

The video registration method showed a decrease in the Rfd values, which was not surprising considering the changes in the chloroplast ultrastructure. The Rfd values, also called the “vitality index”, were shown to be more sensitive and suitable for the assessment of plant stress and vitality than traditionally studied values of quantum yields of photosystem II in a dark-adapted state (Fv/Fm) [52]. Reflecting PSII activity (as well as Fv/Fm), this parameter significantly better correlates with the photosynthetic CO_2_ assimilation than Fv/Fm and other fluorescence-based parameters [47]. In addition, the reason to apply such a method was its ability to obtain photo-induction curves simultaneously from a few leaves, some of them belonging to the untreated plants and some to the NP-treated plants. The method also allowed for decreasing the possible experimental errors. The present results show that the photosynthetic processes in *H. sativum* leaves, at least some PSII functions, are affected by NPs. In addition, it was also found that the NPs affected stomatal aperture and trichome morphology. The observed stomatal dysfunction may affect the regulation of the CO_2_ in-flow [53] which, in turn, aggravates the dysfunction of the PSII activity. These results are consistent with other recent results [32], in which the chlorophyll fluorescence kinetics were evaluated by another parameter—Fv/Fm. With this connection, it is necessary to note that chronic exposure to high levels of ZnO NPs decreased the chlorophyll content and photosynthetic assimilation in *P. australis* [54], and severely affected the growth, chlorophyll content, and Fv/Fm of aquatic plants, viz. *Hydrilla verticillate*, *Azolla filiculoides*, and *Lemna minor* [55,56]. Metal-based NPs are illustrated to damage PS II reaction centers and decrease electron transport [57,58]. Similar observations in our study suggest the phytotoxicity of ZnO NPs.

The ultrastructural modifications in *H. sativum* leaf cells are extended information on what has been previously noted: ZnO NPs induced vacuolation in the root cortical cells of *Lolium perenne* [59] and ruptured the plasma membrane and shrinkage of the protoplast in *A. cepa* [35]. The present results show that the excess content of Zn in the treatments disorganized thylakoids, increased inter-thylakoidal gaps, caused large starch granules, and damaged the inner and outer membranes of the chloroplast. The cytomorphometric analysis indicated a decrease in the sizes and numbers of chloroplasts and plastoglobules per leaf cell, however, the starch granules were visible in the ZnO NP-treated leaf cells. Thylakoids play an important role in lipid metabolic pathways and plastoglobules are its sub-compartment [60]. The changes in plastoglobule sizes and numbers are associated with the stress of the high content of NPs [61], confirming our hypothesis concerned with the toxicity of ZnO NP in plants.

The data on the changes in the ultrastructure of leaf cell organelles obtained in the present research are consistent with the results obtained by other authors [62,63,64]. The degradation of stromal thylakoids was also reported recently [34,65]. In addition, a decrease in the thylakoid number in the grana and swelling of stromal thylakoids upon exposure to heavy metals was noted in the leaves of *Phragmites australis* [63]. The ultrastructure of chloroplast disturbances in the presence of heavy metals is one of the primary reasons for a decrease in the content of the pigment in plants and, in general, for a decrease in the intensity of photosynthesis [66]. Since chloroplasts and mitochondria are the main organelles of photosynthesis, the destructive changes in these organelles after being challenged with ZnO NP treatment are associated with a decrease in the level of metabolic processes that ensure plant growth. Our observations also support the toxicity of ZnO NPs in photosynthetic machinery.

Under the action of high concentrations of Cd, Ni [58], and Cu [59] in plants of different species, the number of plastoglobules increases, indicating the induced degradation of organelles [66,67]. An increase in the number and size of plastoglobules has been observed in the chloroplasts of *Salix purpurea* and *P. australis* exposed to Cd and Cu [68]. A significant increase in the number of plastoglobules is probably due to changes in the membrane structure of plastids. Furthermore, it has been reported that the constituent components of photosynthetic membranes accumulate lipids, proteins, and pigments. These substances are released during the rearrangement of granules under the action of extreme factors, causing reorganization of the plastoglobules [28]. The metal-based NPs may be cytotoxic, genotoxic, and may cause cell cycle arrest and apoptotic induction as well as serious damage to the DNA [28,35]. The release of Zn from ZnO NPs may be the plausible reason for observed changes in this investigation.

## 5. Conclusions

All the tested concentrations of ZnO NPs showed toxic effects and affected the photosynthetic efficiency, anatomy, and ultrastructure of cellular and sub-cellular organelles; the treatment led to disrupted thylakoids, alterations in the inter-thylakoidal gaps, plastoglobules, and starch granules. Cytomorphometric quantification revealed a decrease in the chloroplast, chlorenchyma cell size, shape, and density under the influence of ZnO NP exposure. These modifications are due to the high accumulation of Zn in the leaf tissues and can be considered a defense mechanism against stress induced by ZnO NPs, as this organelle is the main storage site for cytotoxic materials. The present work enhances the mechanistic understanding to improve NPs’ impacts on energy-carrier molecule generation, carbon fixation, and photosynthetic activities without damaging the cellular organization. To the best of our knowledge, this is the first report describing structural modifications of photosynthetic apparatus with photosynthetic activities, destructive and non-destructive quantification of Zn accumulation, and stomatal and trichome morphology in *H. sativum* leaf tissues.

## Figures and Tables

**Table 1:** The accumulation of Zn in above-ground tissues of *H. sativum*, mg/kg.

Treatments	Above-Ground Tissues
Control	21.9 ± 2.1
300 mg/L ZnO NPs	84.2 ± 6.9
2000 mg/L ZnO NPs	224.6 ± 17.3

**Table 2:** The average chlorenchyma cell area, shape, and ratio of chlorenchyma cell area to total leaf cut area, and stomata and trichome area in *H. sativum*.

Treatments	Average Chlorenchyma Cell Area, µm^2^	Shape of Chlorenchyma			Ratio of Chlorenchyma Cell Area to Total Leaf Cut Area	Stomatal	Trichomes
		Circular	Round	Solid			
Control	131 ± 12	0.88	0.74	0.99	0.57	32.0 ± 1.2	12.4 ± 1.5
300 mg/L ZnO NPs	146 ± 14	0.77	0.65	0.95	0.62	39 * ± 1.7	18 * ± 2.6
2000 mg/L ZnO NPs	138 ± 15	0.69	0.58	0.89	0.64	30 ± 1.8	10 ± 1.1

* Reliably differs from the control at *p* < 0.01. Note: Circularity: 4π × [Area]/[Perimeter]^2^ with a value of 1.0 indicates a perfect circle. As the value approaches 0.0, it indicates an increasingly elongated shape. Roundness: 4 × [Area]/π × [Major axis]^2^ isodiametric shape, equal size in all directions. Solidity: [Area]/[Convex area] the presence of invaginations. A value of 1 is a perfect circle.

**Table 3:** Sizes and numbers of chloroplasts, plastoglobules, and starch granules per cell.

Treatments	Size Chloroplasts, µm^2^	Number of Chloroplasts per Cell	Plastoglobules per Cell	Starch Granules per Cell
Control	7.42 ± 0.72	6 ± 0.6	16 ± 3	n/d
300 mg/L ZnO NPs	5.25 ± 0.70	5 ± 0.7	12 ± 2	3.0 ± 0.2
2000 mg/L ZnO NPs	4.17 ± 0.57	4 ± 0.5	18 ± 4	7.1 ± 0.3

## Data Availability

Not applicable.

## Supplementary Materials

The following are available online at https://www.mdpi.com/article/10.3390/nano11071722/s1, X-ray Fluorescence Measurements, Table S1: Net element concentration, wt. % ± one sigma error, Table S2: Atom element concentration, at. %; Figure S1: Optical microscopy image of the measured area and XRF spectra for the control sample, Figure S2: Optical microscopy image (top) of the measured area and XRF spectra (bottom) for the 300 mg/L ZnO NPs sample, Figure S3: Optical microscopy image (top) of the measured area and XRF spectra (bottom) for the 2000 mg/L ZnO NPs sample).
