# Supplementary material for: Effects of Zinc Oxide Nanoparticles on Physiological and Anatomical Indices in Spring Barley Tissues
Source: Nanomaterials (Basel). 2021 Jun 30;11(7):1722. doi: 10.3390/nano11071722 (PMC8307126; doi:10.3390/nano11071722)
Supplement: Supplementary file 1 [file nanomaterials-11-01722-s001.zip › nanomaterials-1247461-supplementary.pdf]

Supporting Information

Effects of Zinc Oxide Nanoparticles on Physiological and Anatomical Indices in Spring Barley Tissues

Vishnu D. Rajput[1] *, Tatiana Minkina[1], Aleksei Fedorenko[1], Natalia Chernikova[1], Tara Hassan[1], Saglara Mandzhieva[1], Svetlana Sushkova[1], Vladimir Lysenko[1], Mikhail A. Soldatov[2] and Marina Burachevskaya[1]

[1]Academy of Biology and Biotechnology, Southern Federal University, Rostov-on-Don 344090, Russia; tminkina@mail.ru (T.M.);
afedorenko@mail.ru (A.F.); nat.tchernikova2013@yandex.ru (N.C.); tara.hassan@su.edu.krd (T.H.); msaglara@mail.ru (S.M.); terra_rossa@mail.ru (S.S.); vs958@yandex.ru (V.L.); marina.0911@mail.ru (M.B.)
[2]The Smart Materials Research Institute, Southern Federal University, Rostov-on-Don, 344090, Russia; mikhail.soldatov@gmail.com (M.A.S.)
* Correspondence: rajput.vishnu@gmail.com; rvishnu@sfedu.ru (V.D.R.); Tel.: +7-918-589-00-93

X-ray Fluorescence Measurements

Non-destructive elemental analysis was performed by means of X-ray fluorescence spectroscopy (XRF). The XRF spectra were measured using microfocus spectrometer M4 Tornado (Bruker Nano GmbH, Berlin, Germany). Rh X-ray tube was operated at 50 kV and 600 μA. The samples were measured in vacuum in order to have better statistics for light elements (Si, P, S, Cl). The data was collected in three different areas for control sample and for two areas for each of the samples that were prepared from the leaves of the plant, treated with 300 mg/L and 2000 mg/L ZnO NPs. For each area 200 points were selected for spectra acquisition. The spectra were analyzed using basic fundamental parameter method. X-ray emission lines of such elements as C, N, O, F, Na are not detectable with this setup and thus are not taken into account in element composition analysis. One sigma error was estimated for each dataset collected in one area.

Initially, 200 scans in different points of one area were measured. While the differences in element concentrations were noticeable from point to point, the one sigma error for 200 scans dataset were ≤0.03 wt.% for K and ≤0.01 wt.% for other elements. Another area with 200 independent points were measured and mean element concentrations were calculated for this dataset as well (for control sample the datasets for 3 areas were collected). Control sample shows moderate reproducibility of element composition from area to area. However, such elements as P, Ca, S, and particularly Si showed significant variation of mean concentration. On the contrary, Zn, Cu, and Fe mean concentration didn't show large variations. The 300 mg/L sample shows particularly high variation of element concentration. In the first area the K concentration significantly drops down, that brings large variation in elemental concentration ratio. This results in the increase of element concentrations of other elements. The second area of this sample shows an increase in concentration of Zn and S. 2000 mg/L sample also shows moderate increase in Zn concentration in both areas.

Presented results of micro-focus XRF measurements showed inhomogeneity in the element distribution throughout the leave, particularly for 300 mg/L sample (Table S1). While the variation of the mean element concentration in different areas of the same sample are quite high, the increase of Zn concentration is clearly visible. Particularly this could be described in terms of concentration ratio between Zn and such plants abundant elements as K, Ca, P, S, and Cl, as well as trace metals Cu and Fe. The increase of Zn element concentration in 300 mg/L and 2000 mg/L samples could imply the presence of Zn-containing particles or just higher Zn concentration in this area. A study of the origin of higher Zn concentration and the fate of ZnO NPs in plants may require more complicated techniques as combined μXRF-μXANES studies that are available at synchrotron.

Table S1. Net element concentration, wt. % ± one sigma error*.

	Control sample			300 mg/L ZnO NPs		2000 mg/L ZnO NPs	
Element	Area 1	Area 2	Area 3	Area 1	Area 2	Area 1	Area 2
K	70.92 ± 0.01	77.00 ± 0.03	72.54 ± 0.02	22.50	66.08	71.67 ± 0.02	84.27 ± 0.02
Cl	17.82	17.94 ± 0.01	20.48 ± 0.01	62.77	20.70	14.60	10.12
P	7.15	3.86	3.97	-	6.94	4.95	4.43
Ca	0.64	0.51	1.33	4.48	1.60	1.65	-
S	1.50	0.51	0.77	0.25	3.86	1.45	0.75
Si	1.77	0.08	0.83	5.64	0.15	5.30	0.19
Fe	0.12	0.06	0.12	1.95	0.14	0.19	0.04
Cu	0.04	0.02	0.03	2.07	0.09	0.05	0.06
Zn	0.04	0.03	0.04	0.33	0.44	0.13	0.14

*Unless otherwise stated, one sigma error was <0.01 wt. % for all measurements

Table S2. Atom element concentration, at. %.

	Control sample			300 mg/L ZnO NPs		2000 mg/L ZnO NPs	
Element	Area 1	Area 2	Area 3	Area 1	Area 2	Area 1	Area 2
K	67.77	74.79	70.08	21.01	63.19	68.24	82.34
Cl	18.78	19.22	21.82	64.64	21.83	15.33	10.91
P	8.62	4.73	4.84	-	8.38	5.95	5.47
Ca	0.60	0.48	1.25	4.08	1.49	1.53	-
S	1.75	0.61	0.77	0.28	4.51	1.69	0.89
Si	2.35	0.11	1.11	7.33	0.19	7.03	0.25
Fe	0.08	0.04	0.08	1.27	0.10	0.13	0.03
Cu	0.02	0.01	0.02	1.19	0.05	0.03	0.03
Zn	0.02	0.02	0.02	0.19	0.25	0.07	0.08
Total				100%			

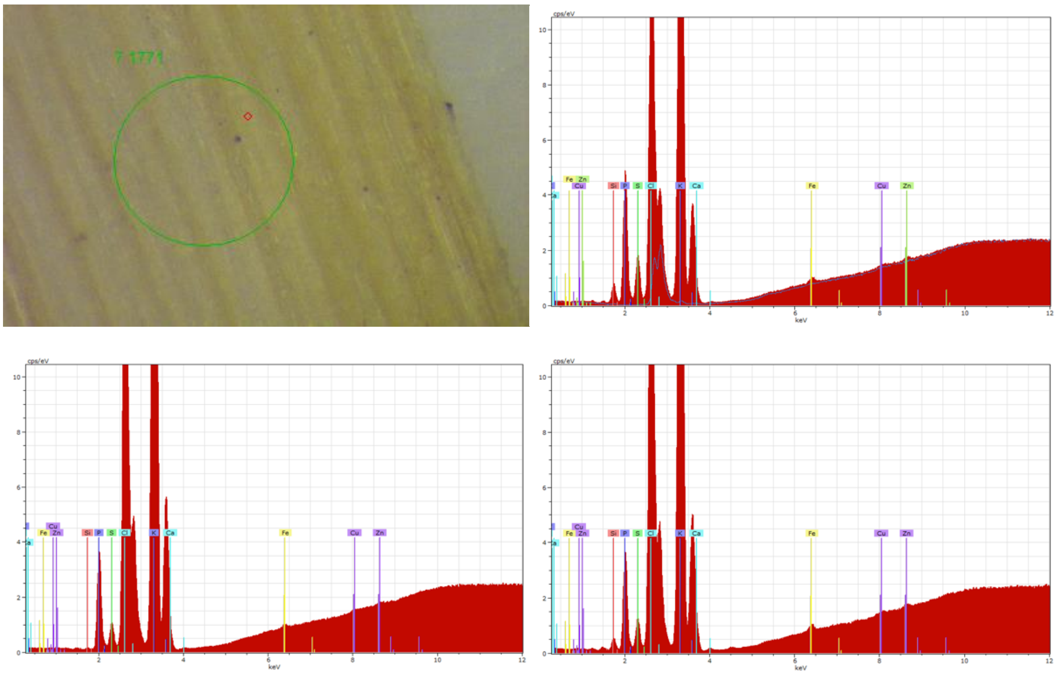

Figure S1. Optical microscopy image of the measured area and XRF spectra for the control sample.

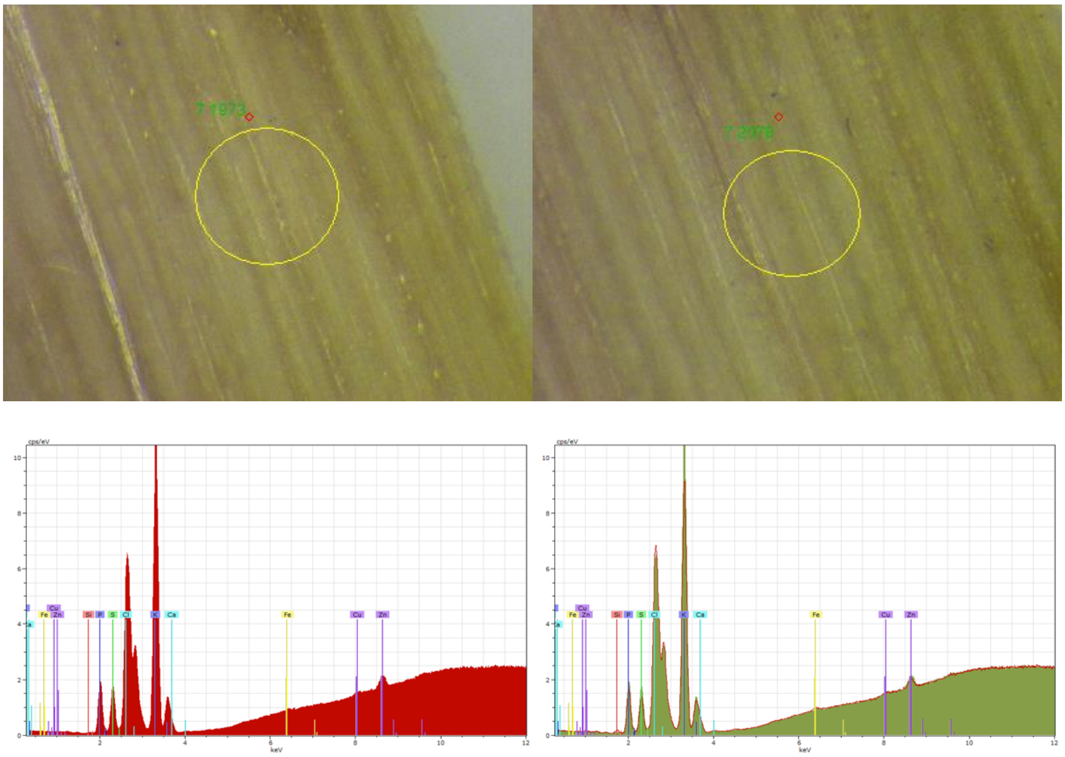

Figure S2. Optical microscopy image (top) of the measured area and XRF spectra (bottom) for the 300 mg/L ZnO NPs sample.

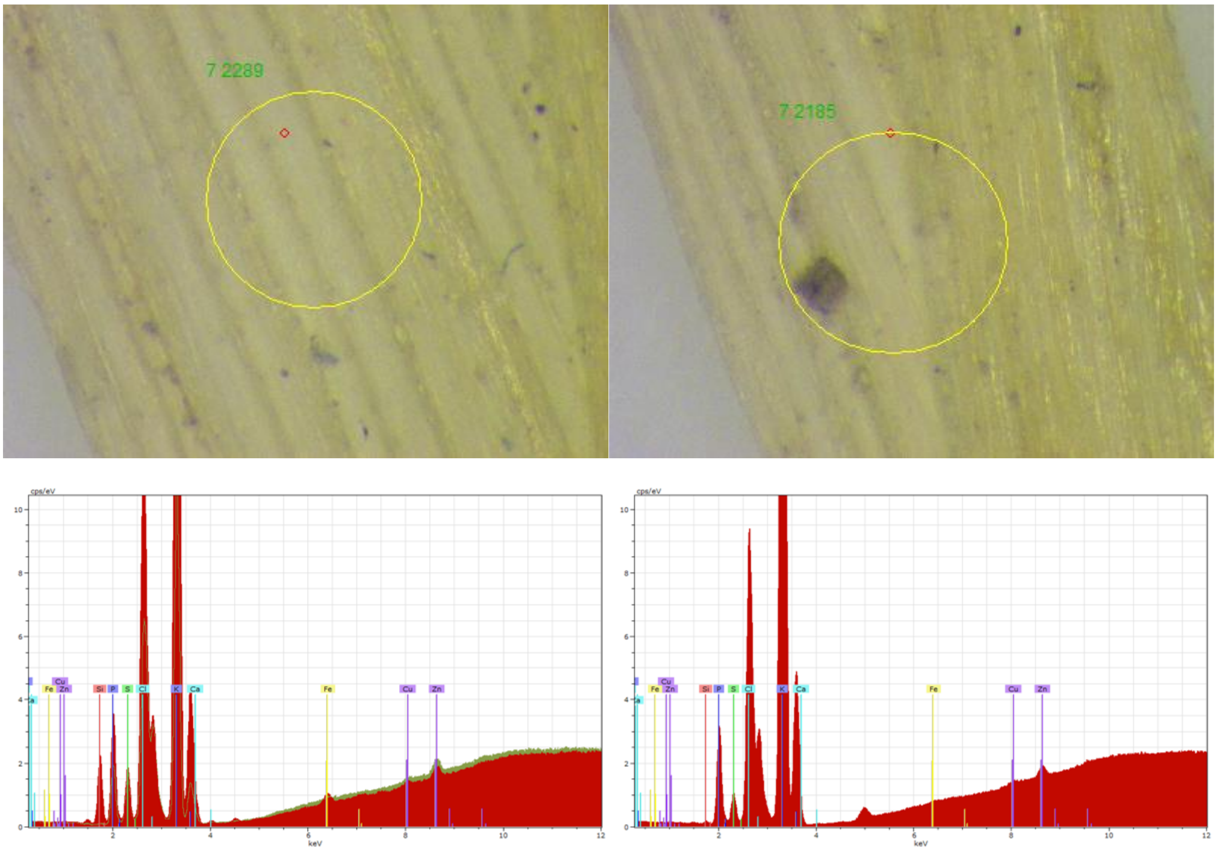

Figure S3. Optical microscopy image (top) of the measured area and XRF spectra (bottom) for the 2000 mg/L ZnO NPs sample.
